# Myoglobin promotes macrophage polarization to M1 type and pyroptosis via the RIG-I/Caspase1/GSDMD signaling pathway in CS-AKI

**DOI:** 10.1038/s41420-022-00894-w

**Published:** 2022-02-28

**Authors:** Ning Li, Jiale Chen, Chenhao Geng, Xinyue Wang, Yuru Wang, Na Sun, Pengtao Wang, Lu Han, Zizheng Li, Haojun Fan, Shike Hou, Yanhua Gong

**Affiliations:** 1 Wenzhou Safety (Emergency) Institute, Tianjin University, Wenzhou, 325000 China; 2 Institute of Disaster and Emergency Medicine, Tianjin University, Tianjin, 300072 China; 3 State Key Laboratory of Medicinal Chemical Biology, Nankai University, Tianjin, 300350 China; 4 Tianjin Key Laboratory of Disaster Medicine Technology, Tianjin, 300072 China; 5 Department of Intensive Care Unit, Tianjin First Center Hospital, Tianjin, 300192 China

**Keywords:** Cell death and immune response, Acute inflammation

## Abstract

Crush syndrome (CS) is a life-threatening illness in traffic accidents and earthquakes. Crush syndrome-induced acute kidney injury (CS-AKI) is considered to be mainly due to myoglobin (Mb) circulation and deposition after skeletal muscle ruptures and releases. Macrophages are the primary immune cells that fight foreign substances and play critical roles in regulating the body’s natural immune response. However, what effect does myoglobin have on macrophages and the mechanisms involved in the CS-AKI remain unclear. This study aims to look into how myoglobin affects macrophages of the CS-AKI model. C57BL/6 mice were used to construct the CS-AKI model by digital crush platform. Biochemical analysis and renal histology confirmed the successful establishment of the CS-AKI mouse model. Ferrous myoglobin was used to treat Raw264.7 macrophages to mimic the CS-AKI cell model in vitro. The macrophage polarization toward M1 type and activation of RIG-I as myoglobin sensor were verified by real-time quantitative PCR (qPCR), Western blotting (WB), and immunofluorescence (IF). Macrophage pyroptosis was observed under light microscopy. The interaction between RIG-I and caspase1 was subsequently explored by co-immunoprecipitation (Co-IP) and IF. Small interfering RNA (siRIG-I) and pyroptosis inhibitor dimethyl fumarate (DMF) were used to verify the role of macrophage polarization and pyroptosis in CS-AKI. In the kidney tissue of CS-AKI mice, macrophage infiltration and M1 type were found. We also detected that in the cell model of CS-AKI in vitro, ferrous myoglobin treatment promoted macrophages polarization to M1. Meanwhile, we observed pyroptosis, and myoglobin activated the RIG-I/Caspase1/GSDMD signaling pathway. In addition, pyroptosis inhibitor DMF not only alleviated kidney injury of CS-AKI mice but also inhibited macrophage polarization to M1 phenotype and pyroptosis via the RIG-I/Caspase1/GSDMD signaling pathway. Our research found that myoglobin promotes macrophage polarization to M1 type and pyroptosis via the RIG-I/Caspase1/GSDMD signaling pathway in CS-AKI.

## Introduction

Crush syndrome (CS), also known as traumatic rhabdomyolysis, refers to the physiological changes that occur after heavy objects are pressed on the skeletal muscle for a long time that usually occurs in traffic accidents and earthquake-related accidents [1, 2]. CS-AKI is a major life-threatening complication that often requires intensive care [3]. Current treatments for CS-AKI are limited to symptomatic treatment and lack specific treatment [4, 5], so CS-AKI poses a high morbidity and mortality rate [6]. Currently, myoglobin is considered to be the main pathogenic factor. The damage to the skeletal muscle occurs when a heavy object crushes it. Then muscle ruptures and releases breakdown products myoglobin into the bloodstream, which can cause toxic effects and tubular obstruction in the kidney [7].

Macrophages are the main components of the immune system that fight foreign substances and play critical roles in regulating the body’s natural immune response [8]. The macrophages in different microenvironments are different and show considerable heterogeneity [9]. They can be classified into two major categories based on their phenotypes and secreted cytokines: pro-inflammatory M1 and anti-inflammatory M2 macrophages [10, 11]. Previous researches indicated that macrophages are the cell type that accumulates in the kidney when tubular cell death or tubule repair in drug and ischemia-induced AKI [12]. However, what effect does myoglobin have on macrophages, and the mechanisms involved in CS-AKI remain unclear yet.

Pyroptosis is a type of programmed cell death that occurs when multiple gasdermin family members are sheared and multimerized, forming gasdermin pores in the plasma membrane [13]. The N-GSDMD terminus binds to inner leaflet lipids of the plasma membrane and multimerizes to generate 10–15 nm diameter pores that trigger the release of cellular contents and inflammatory response [14]. Pyroptosis was first identified in infected macrophages in 1992 [15]. Researchers investigated that pyroptosis may play a mixed blessing role in the pathogenesis and treatment of the disease [16]. Nevertheless, the role of pyroptosis in macrophages in CS-AKI is not clear. Caspase1 is activated by recognition of RNA viruses by RIG-I, causing the release of interleukin-1β to occur [17]. Our previous work indicated that RIG-I could act as a myoglobin sensor to activate NF-κB/Caspase3 signaling pathway in renal tubular epithelial cells in CS-AKI [18]. In addition, several studies have shown that RIG-I is associated with macrophage polarization in viral infections and cancer [19, 20]. However, the role of the RIG-I/Caspase1/GSDMD pathway in macrophage pyroptosis in CS-AKI is unknown. Therefore, in this study, we applied a new digital crush platform to make the CS-AKI mouse model, and to explore the role of the RIG-I/Caspase1/GSDMD signaling pathway in the macrophage polarization and pyroptosis in vivo and in vitro, aiming to elucidate the mechanism of inflammatory response in the CS-AKI model.

## Materials and methods

### Animal models

Male C57BL/6 J mice (about 20 g, 8–10 weeks) were housed in a pathogen-free environment with a 12 h light/dark cycle and free access to food and water. After randomly divided into each group, the C57BL/6 J mice were anesthetized, and the CS-AKI model was created using the digital crush platform [18]. Control group (NC) was performed without pressure. For the CS group (CS), the double hind limbs of mice were placed under the pressure of 1.5 kg for 16 h. After 6 h of decompression, the mice were anesthetized, and their blood and kidney tissues were collected for subsequent experiments. For later animal treatment experiments, we divided the animal experiment into four groups, namely NC, CS, DMF gavage for 7 days, then added crush treatment group (CS + DMF), and the simple gavage for 7 days group (DMF). Investigators were not blinded to group allocations during the experiment or when assessing the outcome.

### Serum biochemistry

The aorta ventralis blood samples taken from each group were coagulated at 4 °C for 30 min. After centrifugation at 3000 rpm for 15 min, serum was collected in 1.5 mL Eppendorf tubes. Creatine kinase (CK), myoglobin, serum creatinine (SCr), and blood urea nitrogen (BUN) were measured using an automatic biochemical analyzer.

### Histology

Euthanized mice at 6 h after removing 1.5 kg pressure to evaluate their muscles and kidneys. One side of the mouse kidney tissue was taken from each group, and the outer envelope was removed. Then these tissue samples were washed three times with 0.9% saline and fixed in 4% paraformaldehyde. After 24 h of tissue fixation, the tissue was dehydrated and embedded in paraffin. Then the tissue was cut into 4 μm thick sections, treated with hematoxylin-eosin (HE) and periodate Schiff (PAS) staining. The pathological changes of the tissue were observed and evaluated under an optical microscope.

### Immunofluorescence and immunohistochemistry

After dewaxing and rehydration, paraffin sections were incubated with 3% hydrogen peroxide, heated in the microwave for antigen repair, and closed with goat serum. For immunohistochemical analysis, sections were labeled with primary antibodies against myoglobin (1:100, Santa Cruz, #sc-74525), F4/80 (1:100, Santa Cruz, #sc-377009), CD86 (1:100, Santa Cruz, #sc-28347), CD206 (1:100, Santa Cruz, #sc-376108), NGAL (1:200, Affinity, #DF6816), and then incubated with horseradish peroxidase-coupled secondary antibodies at 37 °C for 1 h. Sections were then DAB-stained, and nuclei re-stained with hematoxylin, and the samples were visualized by light microscopy. For immunofluorescence analysis, sections labeled with primary antibodies against F4/80, iNOS (1:200, Affinity, #AF0199), Arg1 (1:200, Proteintech, #16001-1-AP), GSDMD and N-GSDMD (1:200, Affinity, #AF4012), IL-1β (1:200, Affinity, #AF5103), cleaved-IL-1β (1:200, Affinity, #AF4006), IL-18 (1:200, Affinity, #DF6252) and then incubated with secondary antibodies coupled to Alexa Fluor 488/594 (1:200, ZSGB-BIO, #ZB-0511 and #ZB-0513) at room temperature for 1 h. Nuclei stained with 4’,6-diamidino-2-phenylindole (DAPI), and observed by fluorescence microscopy or confocal microscopy.

For cell samples, Raw264.7 cells were inoculated onto glass coverslips of 24-well plates and fixed with 4% paraformaldehyde for 20 min, then permeabilized with 0.2% Triton X-100 for 20 min and blocked with 5% skim milk powder at room temperature for 1 h. Cells were incubated with anti-myoglobin (1:100), iNOS (1:200), CD86 (1:200), Arg1 (1:200), RIG-I (1:100, Cell Signaling Technology, #3743), Caspase1 (1:100, Affinity, #AF5418), GSDMD (1:200), IL-1β (1:200) primary antibodies overnight at 4 °C, followed by incubation with Alexa Fluor 488/594-coupled secondary (1:200) antibodies at room temperature for 1 h. Nuclei were finally stained with DAPI for 5 min. Cytoskeleton staining was performed with phalloidine-FITC labeling. Images were finally captured using fluorescence microscopy or confocal microscopy.

### Cell culture and RNA interference

Mouse leukemic monocyte-macrophage cell line Raw264.7 (ATCC) were cultured in a DMEM-high glucose medium containing 10% FBS and antibiotics (100 IU/mL penicillin and 100 mg/mL streptomycin) at 37 °C in humidified air with 5% CO_2_. Authentication was confirmed by the suppliers, and by morphology check with light microscopy. Raw264.7 cells were negative for mycoplasma. To mimic the CS-AKI model in vitro, we harvested cells from the exponential growth phase and treated them with a final 200 μM ferrous myoglobin. Small interfering RNA (siRNA) was synthesized by Gene Pharma (China). siRIG-I has a sense sequence of 5′-GCCCAUUGAAACCAAGAAAUU-3′. GP-Transfect-Mate RNAiMAX was used to transfect siRIG-I (100 nM) following the manufacture’ s instructions. PCR and WB analysis verified the silencing efficiency of the target genes.

### Cell viability

Raw264.7 cells were inoculated into 96-well plates at a concentration of 5 × 10^3^ cells/well for 24 h. Cells were treated with graded concentrations of ferrous myoglobin (50, 100, 200, 300, 400, 500, 600, 700, and 800 μM) for 24 h. Then phosphate-buffered saline (PBS) solution was used to wash the cells. Furthermore, CCK-8 solution (YEASEN, #40203ES60) was added to each well and incubated for 1 h, and the optical density (OD) values were detected at 450 nm by Microplate Reader (BioTek, Epoch 2).

### Co-immunoprecipitation

Total proteins of the control group (NC) and 200 μmol/L ferrous myoglobin treatment group (Mb) of Raw264.7 cells were extracted using IP lysis buffer, and incubated overnight at 4 °C with 1 μg monoclonal anti-RIG-I/anti-Caspase1 antibody and 40 μL protein-A magnetic beads, with shaking. A magnet was used to adsorb the beads, and three times the sample was washed in a binding buffer. Moreover, protein blotting analysis was performed using the anti-Caspase1/anti-RIG-I antibody.

### Quantitative real-time PCR (qPCR)

TRIeasy™ LS Total RNA Extraction Reagent (YEASEN, #19201ES60) was used to lyse cells and extract total RNA, and Nanodrop One was used to determine the purity and concentration of RNA. The Hifair^®^ III 1st Strand cDNA Synthesis Kit (gDNA digester plus) (YEASEN, #11139ES10) was used to synthesize cDNA. LightCycler^®^ 96 instrument (Roche) amplified the qPCR reaction with Hieff^®^ qPCR SYBR^®^ Green Master Mix (No Rox) (YEASEN, 11201ES03). The 2^−ΔΔCt^ method was used to calculate relative expression levels. The primers are listed in Supplementary Table 1.

### Western blotting (WB)

Proteins were extracted from cells and kidney tissue using ice-cold RIPA lysis buffer containing protease inhibitors and phosphatase inhibitors. Samples were centrifuged at 12,000 r/min for 20 min at 4 °C, and the supernatant was collected immediately. 30 μg of total protein per sample was separated by SDS-PAGE and transferred them to PVDF membranes. The membranes were blocked with 5% skimmed milk for 2 h at room temperature before incubation with anti-iNOS (1:1000), anti-CD86 (1:500), anti-IL-6 (1:500), anti-IL-1β (1:500), anti-Arg1 (1:5000), anti-IL-10 (1:500), anti-RIG-I (1:1000), anti-Caspase1 (1: 1000), anti-cleaved-Caspase1 (1:1000), anti-NLRP3 (1:1000), anti-GSDMD (1:1000), anti-N-GSDMD (1:1000), anti-cleaved-IL-1β (1:1000), anti-IL-18 (1:500) and anti-β-Tubulin (1:5000) antibodies at 4 °C overnight. After washing membranes with PBS containing 0.1% Tween 20, incubated with HRP-coupled secondary antibodies (1:5000, Sungene Biotech, #LK2003) for 1 h. The Tanon 5200 Multi Detection System was used to image protein bands visualized with the Enhanced ECL Chemiluminescent Substrate Kit (YEASEN, #36222ES60). The Tanon Gel-Pro Analyzer system was used to measure each band’s intensity.

### Flow cytometry

Raw264.7 cells were grouped according to experiments design. 1 × 10^6^ cells were collected by centrifugation and resuspended in PBS for flow cytometry detection. Anti-iNOS and anti-CD86 antibodies were used to detect macrophage polarization, and anti-N-GSDMD was used to detect pyroptosis. Cells were co-incubated with primary antibodies for 30 min at room temperature and washed twice with PBS. Then cells were incubated with Alexa Fluor 488/594-coupled secondary antibodies for 30 min at room temperature, detected using a CytoFLEX flow cytometer (Beckman, USA) and analyzed using the novoexpress software.

### Statistical analysis

Each experiment was repeated at least three times, and continuous variables with normal distribution were expressed as mean ± standard deviation (SD). For two groups, an unpaired two-tailed Student’s *t* test was used. For more than two groups, one-way ANOVA followed by Dunnett’s method of multiple comparative analysis or two-way ANOVA followed by Tukey’s method of multiple comparative analysis were used. Underlying assumptions for these tests, including sample independence, variance equality, and normality were assumed to be met. All measurements were taken from distinct samples, as noted in figure legends, and no data were excluded. Sample sizes were based in standard protocols in the field. The analysis and graphing were implemented using GraphPad Prism 8.0 software. We set three levels of statistical significance (*P < 0.05; **P < 0.01; ***P < 0.001). Please refer to the figure caption for more details.

## Results

### Macrophages in renal tissue of CS-AKI mice polarize towards M1 type

**Fig. 1:**
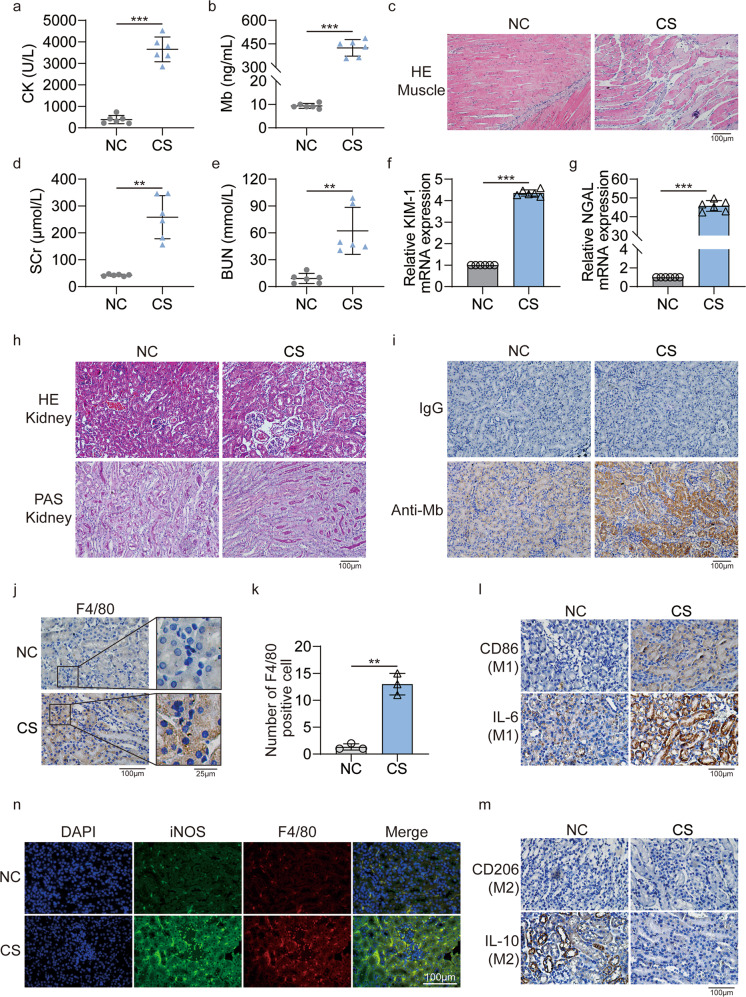
Successful construct the CS-AKI mouse model. **a**, **b** Concentration of biochemical indicators CK and myoglobin in serum. **c** HE staining analyses the pathological changes of muscle in the CS group (original magnification: 200×; scale bar: 100 μm). **d**, **e** Concentration of biochemical indicators SCr and BUN in serum. **f**, **g** qPCR analyses KIM-1 and NGAL mRNA level at the CS group. **h** HE and PAS staining analyze the pathological changes of renal tissues in the CS group (original magnification: 200×; scale bar: 100 μm). **i** IHC staining for myoglobin in the CS group, IgG as a negative control (original magnification: 200×; scale bar: 100 μm). **j** Representative anti-F4/80 staining showing increased macrophage infiltration in CS group compared to NC group (original magnification: 400×; scale bar: 100 μm). **k** F4/80-positive cells are counted equivalent infiltration of macrophages. **l**, **m** IHC staining analyses the expression of M1 associated molecular CD86, IL-6 and M2 associated molecular CD206, IL-10 in kidney tissues (original magnification: 400×, scale bar: 100 μm). **n** Representative confocal microscopy images of sections from kidneys harvested in NC and CS group mice stained for iNOS (green), F4/80 (red) and DAPI (blue) (scale bar: 100 μm). For statistical analysis, an unpaired two-tailed Student’s *t* test was used. Data are expressed as mean ± SD. *n* = 6. **P < 0.01, ***P < 0.001.

The new digital crush platform was used to make the CS-AKI mouse model (Fig. S1a). Serum biochemical indicators showed that compared with the NC group, the concentration of creatine kinase (CK, 383.8 ± 192.2 U/L vs. 3651.0 ± 577.4 U/L, *p* < 0.0001) (Fig. 1a) and myoglobin (9.3 ± 1.0 ng/mL vs. 424.2 ± 52.8 ng/mL, *p* < 0.0001) (Fig. 1b) in the CS group was significantly up-regulated. In the NC group, the muscular tissue has clear horizontal stripes, the cut surface is long, and there are many nuclei. The CS group has apparent muscle damage, including the splitting muscle fibers, rhabdomyolysis, and muscle atrophy (Fig. 1c). The above results indicated that muscle tissue was severely damaged. Meanwhile, biochemical blood tests also showed that compared with the NC group, the concentration of serum creatinine (SCr,42.5 ± 3.5 μmol/L vs. 258.5 ± 80.3 μmol/L, *p* = 0.0012) (Fig. 1d) and blood urea nitrogen (BUN,9.1 ± 5.6 μmol/L vs. 62.3 ± 26.3 μmol/L, *p* = 0.0037) (Fig. 1e) in the CS group was also significantly increased. At the same time, the qPCR results showed that in the kidney tissue of the CS group, the expression of kidney injury molecule 1 (KIM-1) was up-regulated by 4.4 ± 0.1 times (Fig. 1f, *p* < 0.0001), and the expression of neutrophil gelatinase-associated lipocalin (NGAL) was up-regulated by 45.8 ± 2.8 times (Fig. 1g, *p* < 0.0001). The results of HE and PAS showed that the renal tubules in the kidney tissue of the control group had a normal structure. But in the CS group, the results showed tubular necrosis, tubular dilation, cast formation, and infiltration of inflammatory cells (Fig. 1h). Moreover, immunohistochemistry results showed that there were obvious myoglobin casts in the kidney tissues of the CS group (Fig. 1i). Therefore, we successfully constructed a mouse model of CS-AKI.

Immunohistochemistry results showed that there was significant macrophage infiltration in the kidney tissue of the CS group compared with the NC group (Fig. 1j). The statistical results showed that the proportion of F4/80 positive cells in the CS group was increased by 13.0 ± 2.0 times (Fig. 1k, *p* = 0.0062). Macrophages have two distinct phenotypes in different microenvironments: the pro-inflammatory M1 phenotype and the anti-inflammatory M2 phenotype. Immunohistochemistry results indicated that the expressions of M1 macrophage marker CD86 and pro-inflammatory cytokine IL-6 were significantly up-regulated in the kidney tissue of the CS group (Fig. 1l). In contrast, the expression of the M2 surface marker CD206 and the anti-inflammatory cytokine IL-10 was significantly down-regulated (Fig. 1m). Meanwhile, immunofluorescence results also showed that the expression of M1 phenotype marker iNOS in kidney tissue increased (Fig. 1n). Therefore, there is an infiltration of M1 macrophages in the kidney tissue of CS-AKI mice.

### Myoglobin promotes the macrophages polarization into M1 type in vitro

**Fig. 2:**
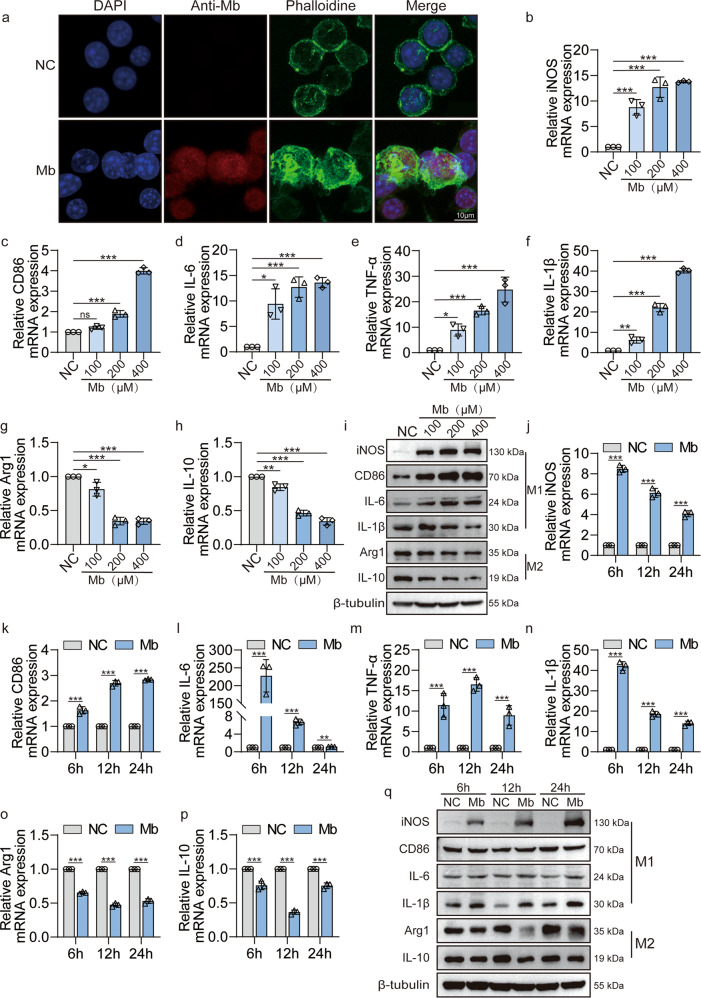
Myoglobin promotes macrophages polarization to M1 phenotype. **a** Representative confocal microscopy images of cells subjected to 200 μM ferrous myoglobin treatments and stained for nuclei (DAPI, blue), anti-myoglobin (red) and microfilament (Phalloidine, green) to detect cytoskeleton (Scale bars: 10 μm). **b**–**i** qPCR and WB analyse the expression of M1 molecules iNOS, CD86, IL-6, TNF-α, IL-1β and M2 molecular Arg1, IL-10 in macrophage that treatment with 100, 200, or 400 μM ferrous myoglobin for 6 h. **j**–**q** qPCR and WB analyse the expression of M1 molecules iNOS, CD86, IL-6, TNF-α, IL-1β and M2 molecular Arg1, IL-10 in macrophage that treatment with 200 μM ferrous myoglobin for 6 h, 12 h and 24 h, respectively. For statistical analysis, one-way ANOVA followed by Dunnett’s method for multiple comparisons were used in (**b**–**h**). Two-way ANOVA followed by Tukey’s method for multiple comparisons used in (**j**–**p**). Data are expressed as mean ± SD, *n* = 3 per group. *P < 0.05, **P < 0.01, ***P < 0.001.

Since we have observed significant myoglobin deposition and M1 macrophage infiltration in the kidney tissue of CS-AKI mice, we speculate that ferrous myoglobin may have an essential effect on macrophage polarization. Therefore, we treated macrophages with ferrous myoglobin in vitro. When activated by antigen stimulation, the morphology changes of macrophages facilitate their phagocytic function. Immunofluorescence results showed that after the treatment with ferrous myoglobin, the skeleton of the macrophages was disordered, indicating that the macrophages were activated (Fig. 2a). The results of CCK8 showed that the IC_50_ value of ferrous myoglobin to the macrophage Raw264.7 was about 564.5 μM (Fig. S2a). Therefore, we used different concentration gradients (100 μM, 200 μM, 400 μM) of ferrous myoglobin below IC_50_ value to treat macrophages. qPCR results revealed that compared with the NC group, the expression of M1 type markers iNOS and CD86 were significantly up-regulated in the Mb group (Fig. 2b, c). Meanwhile, the expression of M1 type related pro-inflammatory cytokines IL-6, TNF-α, and IL-1β also increased (Fig. 2d–f, *p* < 0.0001). The expression of M2 type marker Arg1 and anti-inflammatory cytokine IL-10 were significantly down-regulated (Fig. 2g, h, *p* < 0.0001). WB results showed that the changes at the protein level were consistent with qPCR (Fig. 2i). Therefore, ferrous myoglobin can effectively promote M1 macrophage polarization. Based on the above results and previous literature studies, we chose the concentration of 200 μM ferrous myoglobin for subsequent experiments.

Then macrophages Raw264.7 were treated with 200 μM ferrous myoglobin, qPCR results showed that compared with the NC group, after 6 h, 12 h, 24 h of ferrous myoglobin treatment, the expression of M1 type markers iNOS and CD86 were significantly up-regulated (Fig. 2j, k, *p* < 0.0001). Meanwhile, the expression of M1 type related pro-inflammatory cytokines IL-6, TNF-α, and IL-1β also increased significantly (Fig. 2l–n, *p* < 0.0001). M2 type associate molecules Arg1 and IL-10 expression were down-regulated (Fig. 2o, p). Compared with the NC group, WB results showed that the expression of M1 type associate molecules iNOS, IL-6, and IL-1β at the protein level were consistent with the qPCR results, which significantly up-regulated at 6 h, 12 h, and 24 h, but CD86 did not change significantly. The expression of M2 type associate molecules Arg1 and IL-10 were slightly decreased (Fig. 2q). In addition, immunofluorescence results showed that macrophages treated with ferrous myoglobin for 6 h, 12 h, and 24 h, the expression of M1 marker iNOS increased (Fig. S2b). Moreover, M2 marker Arg1 expression decreased slightly (Fig. S2c). Therefore, ferrous myoglobin can effectively promote the macrophage polarization to M1 type.

### Myoglobin upregulates RIG-I expression and promotes the interaction between RIG-I and Caspase1 in macrophages

**Fig. 3:**
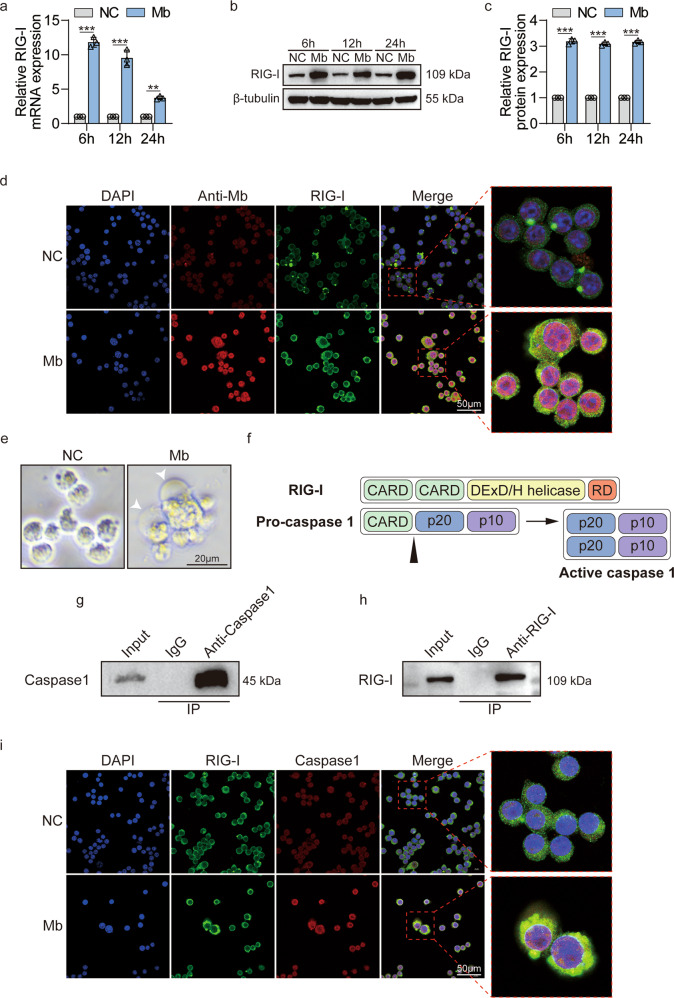
RIG-I is activated by ferrous myoglobin and interacts with caspase1. **a**–**c** qPCR and WB analyse RIG-I expression in macrophage treated with 200 μM ferrous myoglobin for 6 h, 12 h, and 24 h, respectively. **c** is a quantitative analysis of (**b**). **d** Representative confocal microscopy images of cells subjected to 200 μM ferrous myoglobin treatments and stained for nuclei (blue), anti-myoglobin (red), and RIG-I (green) to detect co-localization of myoglobin and RIG-I (Scale bars: 50 μm). **e** Representative cell light microscope images. Arrowheads indicate pyroptotic cells (Scale bars: 20 μm). **f** Schematic diagram of the molecular structure and possible interaction location between RIG-I and caspase1. **g**–**h** Co-IP assays detect the interaction between caspase1 and RIG-I in macrophages that treatment with 200 μM ferrous myoglobin for 12 h. **i** Representative confocal microscopy images of cells subjected to 200 μM ferrous myoglobin treatments and stained for nuclei (blue), RIG-I (green), and caspase1 (red) to detect co-localization of RIG-I and caspase1 (Scale bars: 50 μm). For statistical analysis, two-way ANOVA followed by Tukey’s method for multiple comparisons used in (**a**) and (**c**). Data is expressed as mean ± SD, *n* = 3 per group. **P < 0.01, ***P < 0.001.

Our previous results showed that as damage-associated molecular patterns (DAMPs), ferrous myoglobin could up-regulate the expression of RIG-I in renal tubular epithelial cells [18]. Do we wonder whether the same phenomenon occurs in macrophages? qPCR results showed that after 6 h, 12 h, and 24 h treatment with 200 μM ferrous myoglobin, the expression of RIG-I in macrophages was significantly up-regulated (Fig. 3a). The WB results showed the same trend (Fig. 3b, c). In addition, the immunofluorescence results showed the co-localization of myoglobin and RIG-I (Fig. 3d). Therefore, ferrous myoglobin up-regulates the expression of RIG-I in macrophages.

Meanwhile, during the process of ferrous myoglobin treatment, cell pyroptosis was observed in macrophages (Fig. 3e). Based on our previous RNA sequencing results of kidney tissue in the CS-AKI rat model squeezed with a 3 kg weight, nineteen NOD-like signaling pathway molecules are down-regulated, and eight are up-regulated. Among the up-regulated genes contains the pyroptosis executive molecular GSDMD (Fig. S3a). At the same time, compared with the NC group, the expression of GSDMD was up-regulated in the kidney tissues of the 3 kg and 5 kg weight compression groups (Fig. S3b). Caspase1 plays a vital role in the cleaving of GSDMD into an active form. Meanwhile, both RIG-I and caspase1 have CARD domains (Fig. 3f), so we guess there exist an interaction between RIG-I and caspase1. The results of the co-immunoprecipitation showed that caspase1 interacts with RIG-I (Fig. 3g, h). We further found that after Raw264.7 cells were treated with ferrous myoglobin, RIG-I and caspase1 are co-localization (Fig. 3i). Therefore, ferrous myoglobin promotes the interaction and co-localization between RIG-I and caspase1 in macrophages.

### siRIG-I decreases the myoglobin induced expression of RIG-I and pyroptosis molecules

**Fig. 4:**
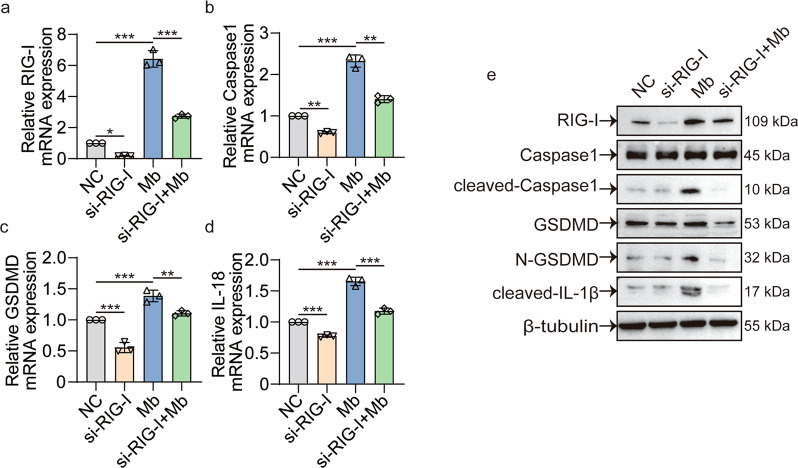
siRIG-I decreases the myoglobin induced expression of RIG-I and pyroptosis molecules. **a**–**d** qPCR analyses RIG-I, caspase1, GSDMD, IL-18 expression in control group (NC), siRIG-I group, ferrous myoglobin treatment group (Mb), and knocking down RIG-I then treatment with ferrous myoglobin group (siRIG-I + Mb). **e** WB analyses RIG-I, caspase1, cleaved-caspase1, GSDMD, N-GSDMD, cleaved-IL-1β expression in NC group, siRIG-I group, Mb group, and siRIG-I + Mb group. For statistical analysis, one-way ANOVA followed by Tukey’s method for multiple comparisons used in (**a**–**d**). Data are expressed as mean ± SD, *n* = 3 per group. *P < 0.05, **P < 0.01, ***P < 0.001.

Next, we used siRIG-I to further verify the relationship between RIG-I and caspase1 mediated pyroptosis pathway in ferrous myoglobin treated macrophages. qPCR and WB results showed that siRIG-I could effectively knock down RIG-I’s expression (Fig. 4a, e). Meanwhile, caspase1 expression in mRNA level and cleaved-caspase1 expression in protein level were significantly decreased (Fig. 4b, e). Compared with the Mb group, knocking down RIG-I and then treating with ferrous myoglobin (siRIG-I + Mb) would down-regulate the expression of pyroptosis-associated molecular GSDMD, N-GSDMD, IL-1β, and IL-18 (Fig. 4b–e). These results confirmed the relationship between RIG-I and caspase1 and indicated that RIG-I plays a vital role in the caspase1 mediated pyroptosis signaling pathway.

### DMF inhibits myoglobin activated RIG-I/Caspase1/GSDMD pyroptosis pathway

**Fig. 5:**
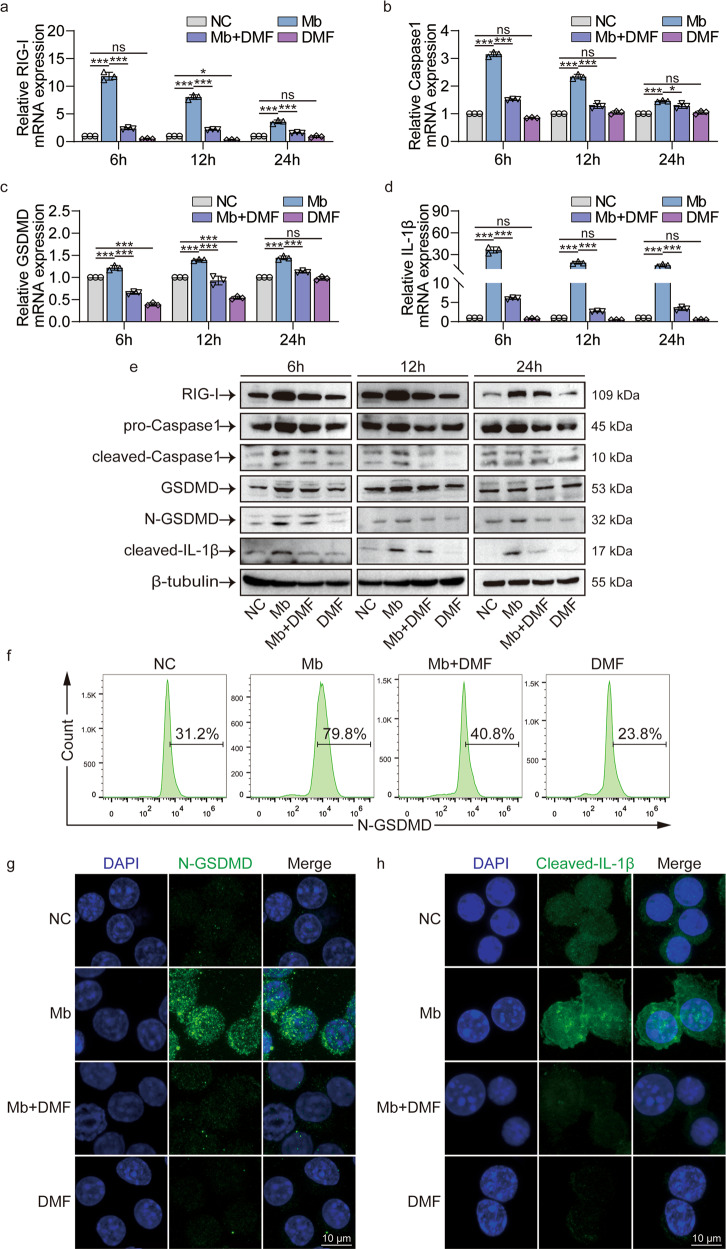
DMF inhibits RIG-I/Caspase1/GSDMD pyroptosis pathway after ferrous myoglobin treatment. **a**–**d** qPCR analyses RIG-I, caspase1, GSDMD, IL-1β expression in NC, Mb, Mb + DMF, and DMF group. **e** WB analyses RIG-I, caspase1, cleaved-caspase1, GSDMD, N-GSDMD, cleaved-IL-1β expression in NC, Mb, Mb+DMF, and DMF group. **f** Flow cytometry analyses N-GSDMD expression in NC, Mb, Mb+DMF, and DMF group. **g**, **h** Representative confocal microscopy images of cells in NC, Mb, Mb + DMF, and DMF group stain for N-GSDMD and cleaved-IL-1β (Scale bars: 10 μm). For statistical analysis, two-factor ANOVA followed by Tukey’s method of multiple comparisons used in (**a**–**d**). Data are expressed as mean ± SD, *n* = 3 per group. *P < 0.05, **P < 0.01, ***P < 0.001.

We use pyroptosis inhibitor DMF to verify the RIG-I/Caspase1/GSDMD pyroptosis pathway’s function in ferrous myoglobin treated Raw264.7 macrophages. qPCR results showed that Raw264.7 cells treatment with ferrous myoglobin for 6, 12, and 24 h showed an increase in the expression of RIG-I and pyroptosis-related molecules caspase1, GSDMD, IL-1β, and IL-18 compared with the NC group (Figs. 5a–d, S4a). Pyroptosis inhibitor DMF treatment then added ferrous myoglobin (Mb + DMF group), the expressions of RIG-I and pyroptosis-related molecules caspase1, GSDMD, IL-1β, and IL-18 were all decreased (Figs. 5a–d, S4a). Compared with the NC group, the pyroptosis-related molecules IL-1β and IL-18 did not change, and the expression of GSDMD was slightly reduced at 6 and 12 h in DMF group (Figs. 5a–d, S4a). WB results showed that the expressions of RIG-I, cleaved-caspase1, N-GSDMD, and cleaved-IL-1β were significantly up-regulated in the Mb group and then significantly down-regulated in the Mb + DMF group compared to NC group (Fig. 5e). Flow cytometry results also showed that the expression of N-GSDMD increased in the Mb group and decreased in the Mb+DMF group (Fig. 5f). Meanwhile, immunofluorescence results showed that, in contrast to the Mb group, the Mb+DMF group decreased expression of N-GSDMD, cleaved-IL-1β, and IL-18 (Figs. 5g, h, S4b). Therefore, DMF inhibits RIG-I/caspase1/GSDMD pyroptosis signaling pathway in macrophages treated with ferrous myoglobin.

### DMF inhibits macrophage polarization to M1 phenotype after treatment with ferrous myoglobin

**Fig. 6:**
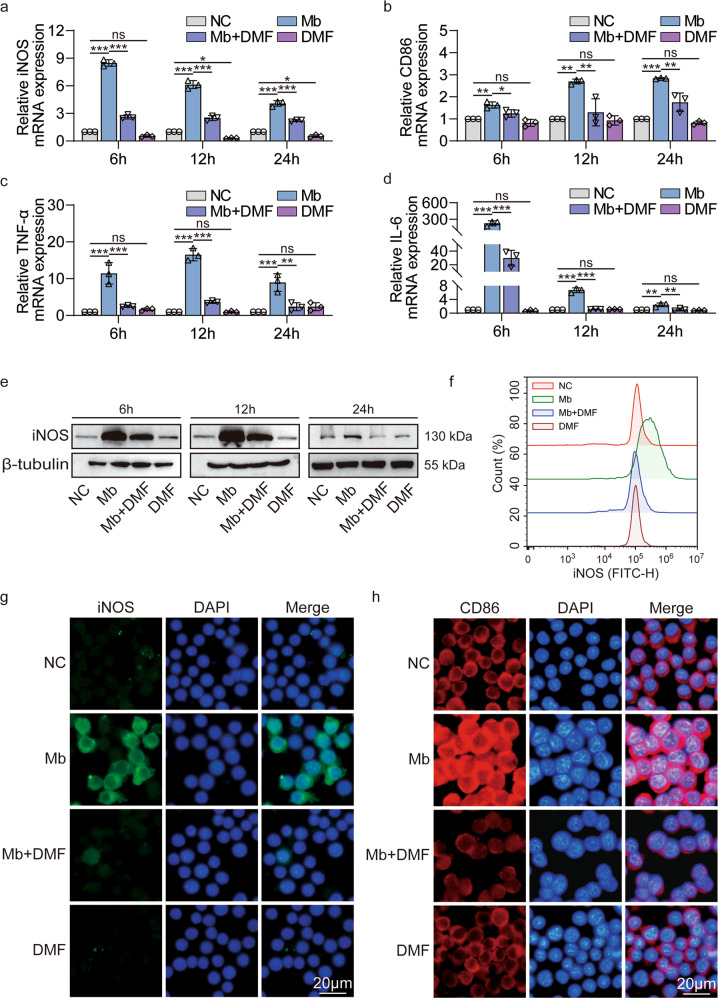
DMF inhibits macrophage polarization to M1 phenotype after treatment with ferrous myoglobin. **a**–**d** qPCR analyses iNOS, CD86, TNF-α, IL-6 expression in NC, Mb, Mb + DMF, and DMF group. **e**, **f** WB and flow cytometry analyse iNOS expression in NC, Mb, Mb+DMF, and DMF group. **g**, **h** Representative confocal microscopy images of cells in NC, Mb, Mb + DMF, and DMF group stained for iNOS and CD86 (Scale bars: 20 μm). For statistical analysis, two-factor ANOVA followed by Tukey’s method of multiple comparisons used in (**a**–**d**). Data are expressed as mean ± SD, *n* = 3 per group. *P < 0.05, **P < 0.01, ***P < 0.001.

DMF inhibits the macrophage pyroptosis caused by ferrous myoglobin, so what is the effect on macrophages polarization? As shown by qPCR results, the levels of M1 markers iNOS, CD86, and pro-inflammatory factors TNF-α, IL-6 were significantly reduced in the Mb+DMF group compared to the Mb group at 6, 12, and 24 h, respectively (Fig. 6a–d). Compared with the NC group, WB and flow cytometry results showed that the expression of M1 markers iNOS was significantly up-regulated in the Mb group at 6, 12, and 24 h (Fig. 6e–f). After DMF treatment, it can effectively inhibit the expression of iNOS (Fig. 6e–f). The immunofluorescence experiment obtained the same trend. The Mb + DMF group showed decreased expression of the M1 biomarker iNOS and CD86 compared with the Mb group (Fig. 6g–h). Therefore, the pyroptosis inhibitor DMF inhibits macrophages polarization to M1 phenotype after treatment with ferrous myoglobin.

### DMF alleviates renal injury in CS-AKI mice by reducing M1 macrophage polarization and pyroptosis

**Fig. 7:**
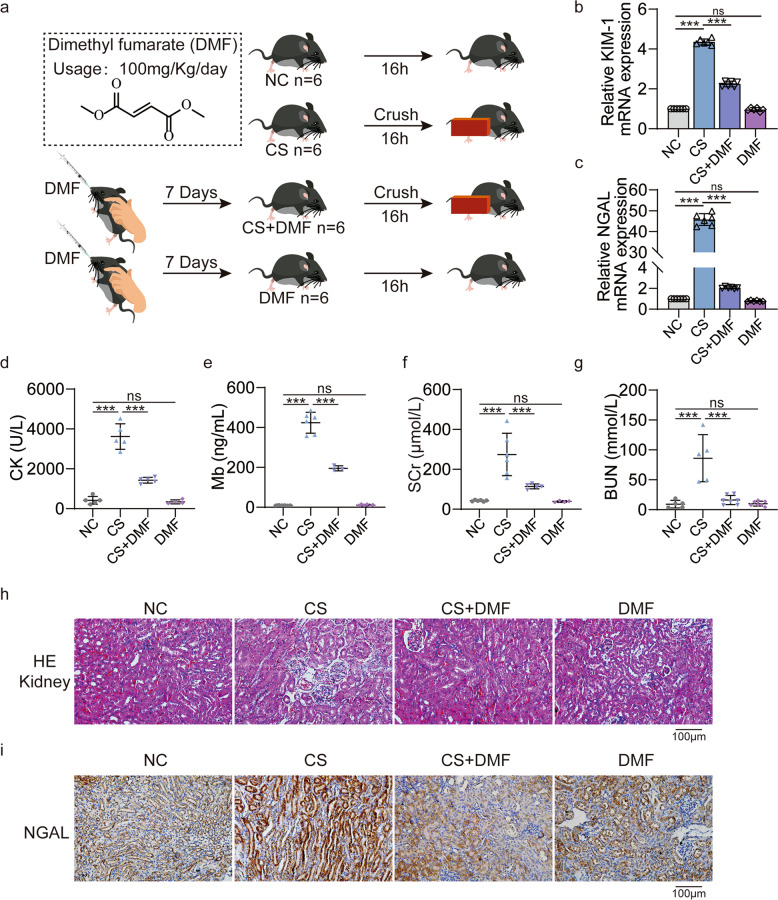
DMF alleviated renal injury in CS-AKI mice. **a** Schematic diagram of experimental design of DMF treatment for CS-AKI mice. **b**, **c** qPCR analyses KIM-1 and NGAL mRNA level in the renal tissues of four groups (NC, CS, CS + DMF, DMF). **d**–**g** Concentration of biochemical indicators CK, myoglobin, SCr and BUN in serum. **h** Evaluation of the therapeutic effect of DMF on the kidney tissue of CS-AKI mice by HE staining (original magnification: 200×, scale bar: 100 μm). **i** IHC staining analyses the expression of NGAL in kidney tissues (original magnification: 200×, scale bar: 100 μm). For statistical analysis, one-way ANOVA followed by Tukey’s method for multiple comparisons used in (**b**–**g**). Data are expressed as mean ± SD, *n* = 3 per group. **P < 0.01, ***P < 0.001.

The grouping (NC, CS, CS + DMF, DMF) of mice and the method of administration are shown in Fig. 7a. Compared with the CS group, qPCR results showed that KIM-1 and NGAL in the kidney tissue of the CS + DMF group were significantly decreased (Fig. 7b, c). At the same time, the CK, myoglobin, SCr, BUN have good consistency with them (Fig. 7d–g). There was no change in kidney injury and biochemical blood indicators in the DMF group compared with the NC group (Fig. 7b–g). Both HE and NGAL immunohistochemistry staining showed that the kidney damage in the CS + DMF group was less than that in the CS group (Fig. 7h, i). Therefore, DMF alleviated kidney damage in CS-AKI mice.

**Fig. 8:**
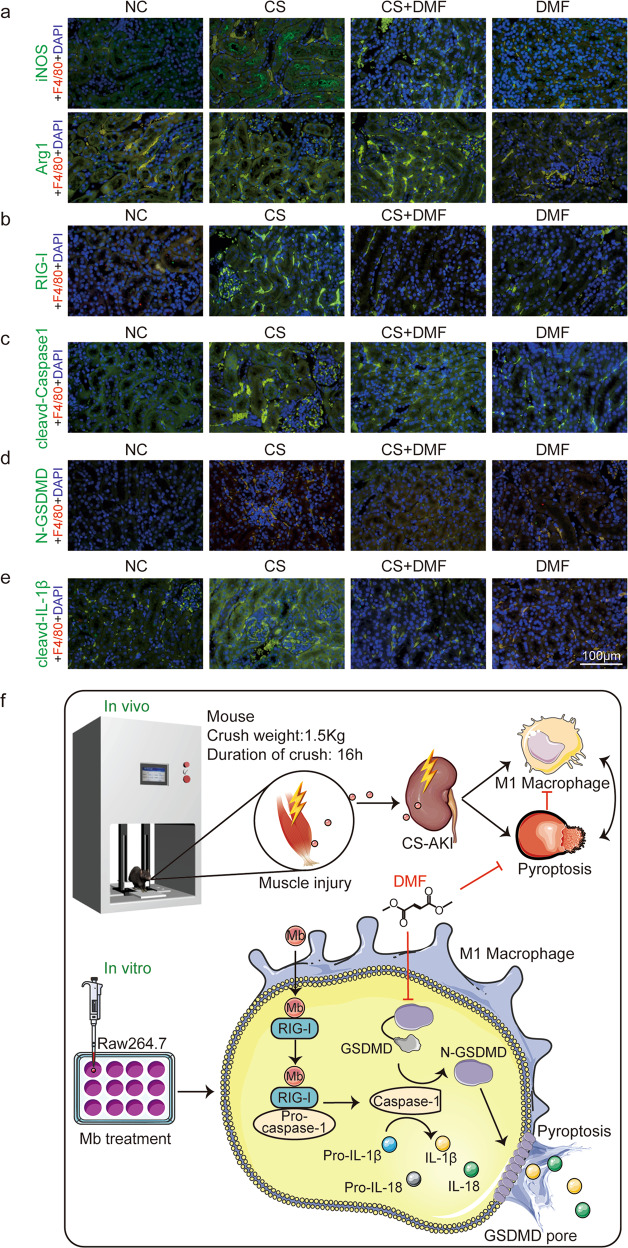
DMF reduces M1 macrophage activation and macrophage pyroptosis. **a**–**e** Representative confocal microscopy images of sections from kidneys harvested in NC, CS, CS + DMF and DMF group mice stained for iNOS, Arg1, RIG-I, cleaved-Caspase1, N-GSDMD, and cleaved-IL-1β (green), F4/80 (red) and DAPI (blue) (scale bar: 100 μm). *n* = 6. **f** The model of myoglobin induced macrophage polarization and pyroptosis in CS-AKI. Mice decompressed after 16 h continuous compression with a 1.5 kg weight have damage to the muscles of their legs, and the necrotic muscles release myoglobin, which reaches the kidneys with the blood circulation and leads to the polarization of the kidney macrophages toward M1 type and pyroptosis. Raw264.7 macrophages treated with ferrous myoglobin to mimic cell model of CS-AKI in vitro promote macrophage polarization and pyroptosis via RIG-I/Caspase1/GSDMD signaling pathway. And promotes mature IL-18 and IL-1β release. Pyroptosis inhibitor DMF can inhibit macrophage polarization and pyroptosis in CS-AKI.

Does DMF reduce kidney damage in CS-AKI mice related to macrophage infiltration and phenotype transformation? In order to answer this question, we performed in situ immunofluorescence staining of kidney tissue sections in different treatment groups. The immunofluorescence results showed that compared with NC group, there was macrophage infiltration (F4/80) in the kidney tissue of the CS group and the infiltration of M1 macrophages (iNOS) increased (Fig. 8a, Upper). At the same time, the M2 type biomarker Arg1 in the CS group had no significant change compared with the NC group (Fig. 8a, lower). Compared with the CS group, the infiltration of M1 macrophages in the CS + DMF group was significantly reduced (Fig. 8a, Upper), and the M2 macrophage increased (Fig. 8a, lower). Therefore, DMF, an inhibitor of pyroptosis, can inhibit the transformation of macrophages to M1 phenotype in the kidneys of CS-AKI mice.

Next, we explored whether reducing kidney damage in CS-AKI mice by DMF is related to the RIG-I/Caspase1/GSDMD pyroptosis signaling pathway? Immunofluorescence results showed that compared with the NC group, the expression of RIG-I in macrophages in the kidney tissue of the CS group was up-regulated (Fig. 8b). Meanwhile, the expression of RIG-I decreased in the CS + DMF group compared with the CS group (Fig. 8b). At the same time, cleaved-caspase1, GSDMD, N-GSDMD, IL-1β, cleaved-IL-1β had the same trend (Figs. 8c–e, S5a, b). Therefore, DMF inhibits the RIG-I/Caspase1/GSDMD pyroptosis pathway of macrophages in the kidney of CS-AKI mice.

## Discussion

Crush syndrome has a similar pathological process to rhabdomyolysis [21]. Crush syndrome is frequently related to acute kidney injury. Unfortunately, there are no specific treatments for CS-AKI and affected patients’ managements are mainly symptomatic [22]. Researches indicated that myoglobin is involved in the pathophysiology of AKI and acts as a molecule of damage-associated molecular patterns (DAMPs) [18]. Myoglobin as a foreign stimulus induces a renal immune response [23]. Macrophage is one of the major immune cells. And the relationship between myoglobin and macrophages has been explored in rhabdomyolysis-induced AKI (RM-AKI) [24–27]. Mesenchymal stem cells (MSCs) can ameliorate RM-AKI by activating macrophages to trophic M2, which supports the transition from tubular injury to tubular repair [26]. In addition, Erythropoietin (EPO) ameliorates renal injury in vivo by reducing macrophage recruitment and promoting phenotypic conversion to M2 macrophages [27]. However, macrophages infiltration, polarization and pyroptosis have been less studied in CS-AKI models.

Therefore, our study explores whether there is macrophage infiltration in the kidney tissue of the CS-AKI mouse model? If so, how does the phenotype of macrophages? Do macrophages undergo pyroptosis? To answer this question, we first use homemade crush platform for preparing CS-AKI mouse model. Compared with other modeling methods, such as rubber band binding, heavyweight crush, and glycerol injection, it is more realistic and accurate [4]. During the squeezing process, the pressure can be mediated by the controller. And when it enters the hold mode, the pressure can be automatically constant in the set range. The pressure value is displayed in real-time, and the speed can be set during descent. Previous studies have used the crush platform to make CS-AKI rat model [18, 28, 29], but this is the first time to use the crush platform to explore the mouse model. The CS-AKI mouse model was constructed by applying 1.5 kg pressure to both lower extremities for 16 h. We also examined the biochemical indicators of serum, kidney injury biomarker, HE and PAS staining of kidney tissues to verify the success of the CS-AKI mouse model.

In the CS-AKI mice, we detected macrophage infiltration in the kidney tissue. We also found macrophage polarization to M1 phenotype and myoglobin deposition in the kidney of CS-AKI mice. Therefore, we next explored the effects of myoglobin on macrophages in detail in a cellular model. One study treated Raw264.7 macrophages with 200 μM myoglobin in the RM-AKI model, which only explored the TLR4/NF-κB pathway and did not investigate macrophage polarization [30]. CCK-8 assay results showed the IC_50_ of ferrous myoglobin on Raw264.7 macrophages was 564.5 μM. To further investigate the effect of myoglobin on macrophages, three concentrations of 100, 200, and 400 μM were used to treat macrophages. After ferrous myoglobin treatment, M1 related molecule expression significantly increased.

RIG-I, also known as Ddx58, is a pathogen pattern recognition receptor, and it has a vital role in innate antiviral immunity [31]. In addition, RIG-I recognizes endogenous RNA, DNA, proteins, and other biomolecules involved in chronic inflammatory diseases, autoimmune diseases, tumors, and other disease processes [32–34]. In non-viral infection studies, our recently research have shown that RIG-I acts as a sensor for myoglobin to activate the RIG-I/NF-κB/Caspase3 signaling pathway, which promotes the secretion of inflammatory factors and mediates apoptosis in renal cells of CS-AKI rat [18]. Therefore, we speculated whether RIG-I expression of macrophages could be up-regulated after myoglobin treatment.

Meanwhile, during the process of ferrous myoglobin treating macrophages, we observed pyroptosis. Pyroptosis is an essential component of the innate immune system [35, 36] and is associated with many immune cells [37–41]. There is also a distinction between classical and non-classical pathways of pyroptosis [42]. Pyroptosis relies on inflammasome to activate some caspase family proteins [43], causing them to cleave the GSDMD, which activates the GSDMD [44]. The activated GSDMD (N-GSDMD) translocate onto the membrane and forms 10~15 nm GSDMD pores [45], cell swelling, and cytoplasm efflux [46]. The gasdermin family includes GSDMA, GSDMB, GSDMC, DFNA5, DFNB59, etc. [47, 48]. One study found that PD-L1 mediated expression of GSDMC converts apoptosis of cancer cells to pyroptosis and promotes tumor necrosis [49]. GSDME deficiency attenuates acute kidney injury by inhibiting pyroptosis and inflammation [50]. In addition, our previous RNA sequencing results showed that after the decompression 12 h kidney tissue of CS-AKI rat, 19 of 27 molecules in the NOD-like signaling pathway were down-regulated, and eight were up-regulated. The up-regulated gene contained the pyroptosis execution protein GSDMD. Meanwhile, considering that both RIG-I and caspase1 have CARD domains, and RIG-I has been linked to caspase1 in viral studies [17]. Therefore, we want to explore the role of RIG-I/Caspase1/GSDMD signaling pathway in the macrophages of CS-AKI mice. Both in vitro and in vivo models showed that myoglobin promotes macrophage pyroptosis through RIG-I/Caspase1/GSDMD signaling pathway in CS-AKI. Meanwhile, pyroptosis inhibitor DMF that targets GSDMD [51], could inhibits RIG-I/Caspase1/GSDMD signaling pathway. This suggests that DMF not only targets GSDMD but also inhibits the entire classical pyroptosis pathway. Interesting, DMF also inhibited the macrophages polarization to M1 type in the kidney tissue of CS-AKI mice. This is consistent with the results of several studies showing that DMF has anti-inflammatory effects [52, 53]. However, contrary to our findings, one study showed that caspase1 attenuates RIG-I mediated signaling activity in poly(I:C) treatment [54]. This may be caused by the different responses of the human immune system to viral infectious diseases and non-infectious diseases.

There are many ways attempt to treat CS-AKI, including EPO therapy, mesenchymal stem cell therapy, alpha-1-acid glycoprotein therapy, etc. [4, 26, 27, 55, 56]. DMF may be a novel therapy for the CS-AKI. However, we only investigated the classical pyroptosis pathway in the CS-AKI model and did not verify whether the non-classical pyroptosis pathway was activated in the CS-AKI model. In addition, heme-activated platelets from necrotic muscle cells enhanced the production of macrophage extracellular traps (METs), which suggested to be a cause of CS-AKI [57], and many studies also found that METs was associated with pyroptosis in the rhabdomyolysis model [40, 58]. We did not explore the relationship between METs and pyroptosis in the CS-AKI model. Our research has not completely resolved whether the macrophage polarization caused the pyroptosis, or the pyroptosis caused the polarization. Macrophage polarization to pyroptosis and pyroptosis to macrophage polarization circles could function in CS-AKI.

In conclusion, we successfully constructed a mouse model of CS-AKI. There is macrophage infiltration and myoglobin deposition in the kidney tissue of CS-AKI mice. Ferrous myoglobin promoted macrophage polarization to M1 type and pyroptosis via the RIG-I/Caspase1/GSDMD signaling pathway in CS-AKI. Meanwhile, pyroptosis inhibitor DMF attenuated kidney injury of CS-AKI mice by reducing M1 macrophage polarization and pyroptosis via RIG-I/Caspase1/GSDMD signaling pathway (Fig. 8f).

## Supplementary information

Original Data File

Supplementary Information

## Data Availability

All data that support the findings of this study are available from the corresponding authors upon reasonable request. All of full and uncropped western blots are shown in Supplementary material of original WB data.
