# Supplementary material for: Myoglobin promotes macrophage polarization to M1 type and pyroptosis via the RIG-I/Caspase1/GSDMD signaling pathway in CS-AKI
Source: Cell Death Discov. 2022 Feb 28;8:90. doi: 10.1038/s41420-022-00894-w (PMC8885737; doi:10.1038/s41420-022-00894-w)

**Supplementary material of original WB data for**

Myoglobin promotes macrophage polarization to M1 type and pyroptosis via the RIG-I/Caspase1/GSDMD signaling pathway in CS-AKI

**The original WB image of Figure 2 and Figure 3**


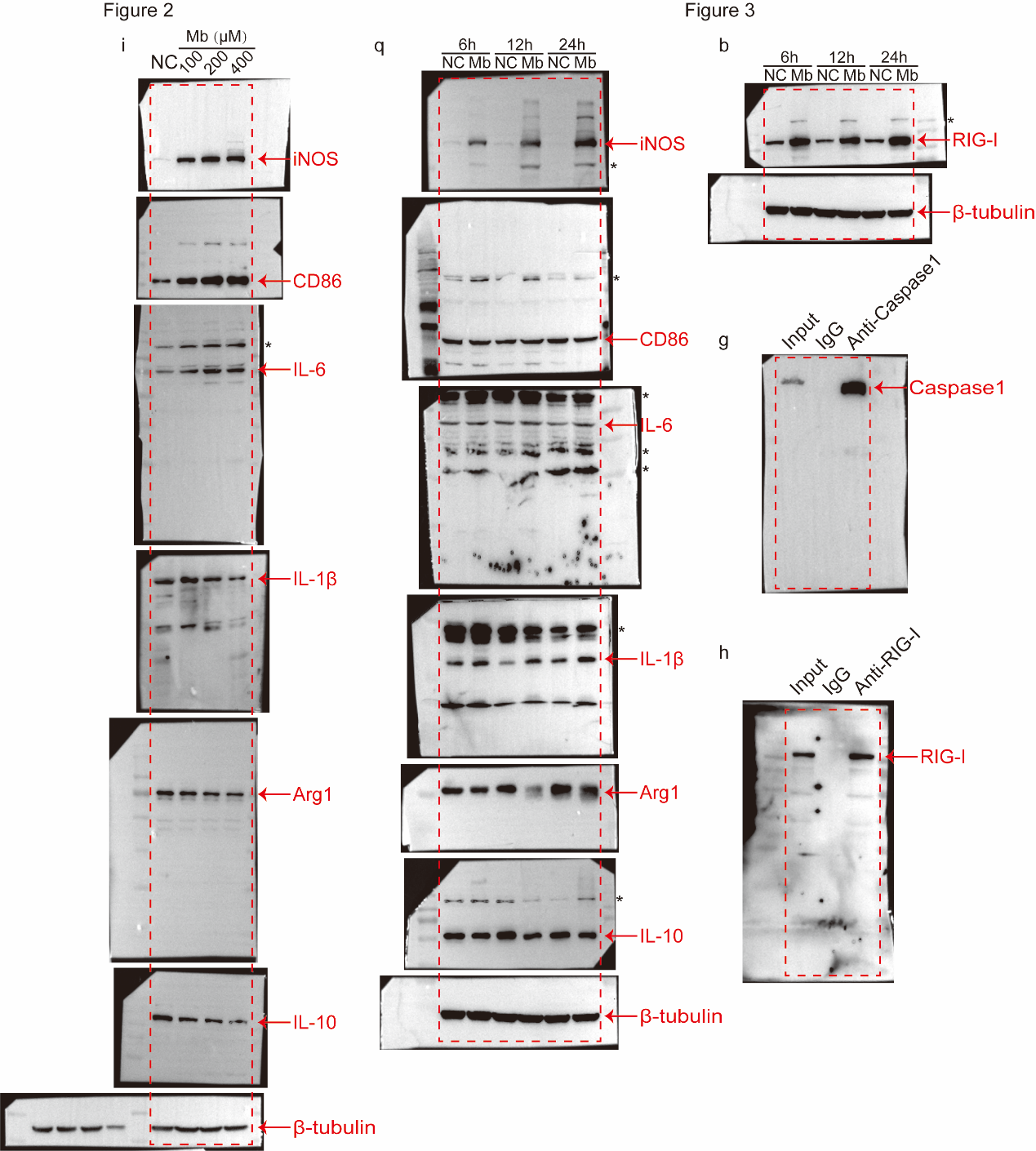


Notes：Gray asterisks denote a non-specific band.

**The original WB image of Figure 4**


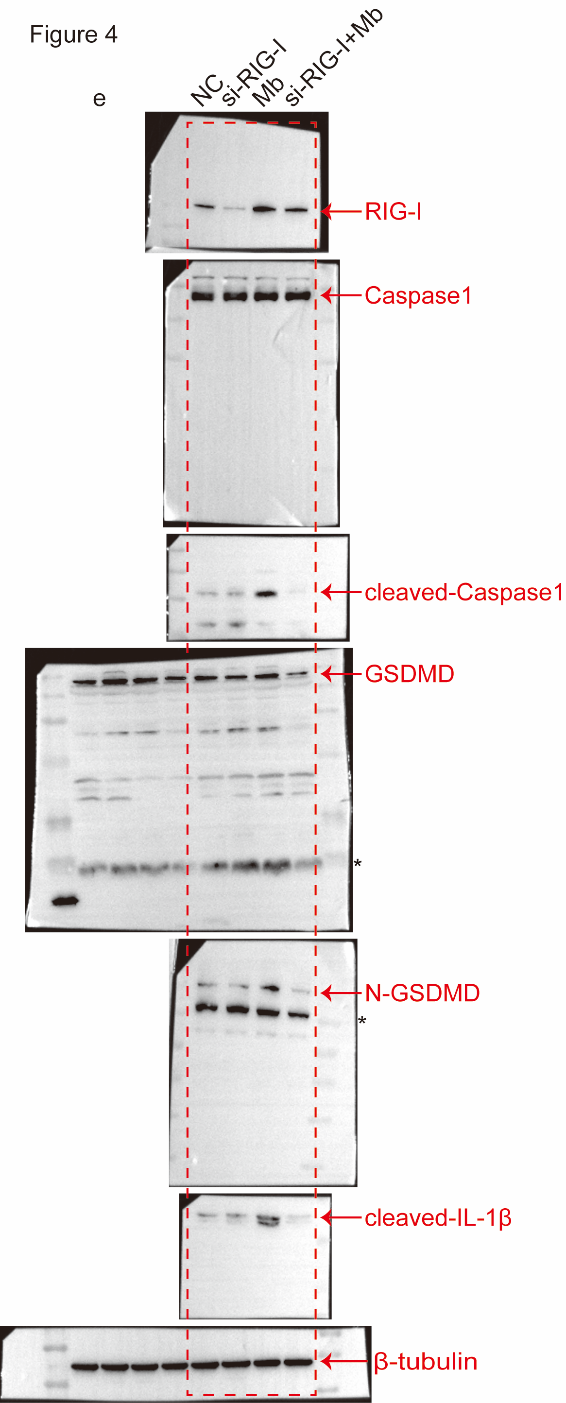


Notes：Gray asterisks denote a non-specific band.

**The original WB image of Figure 5**


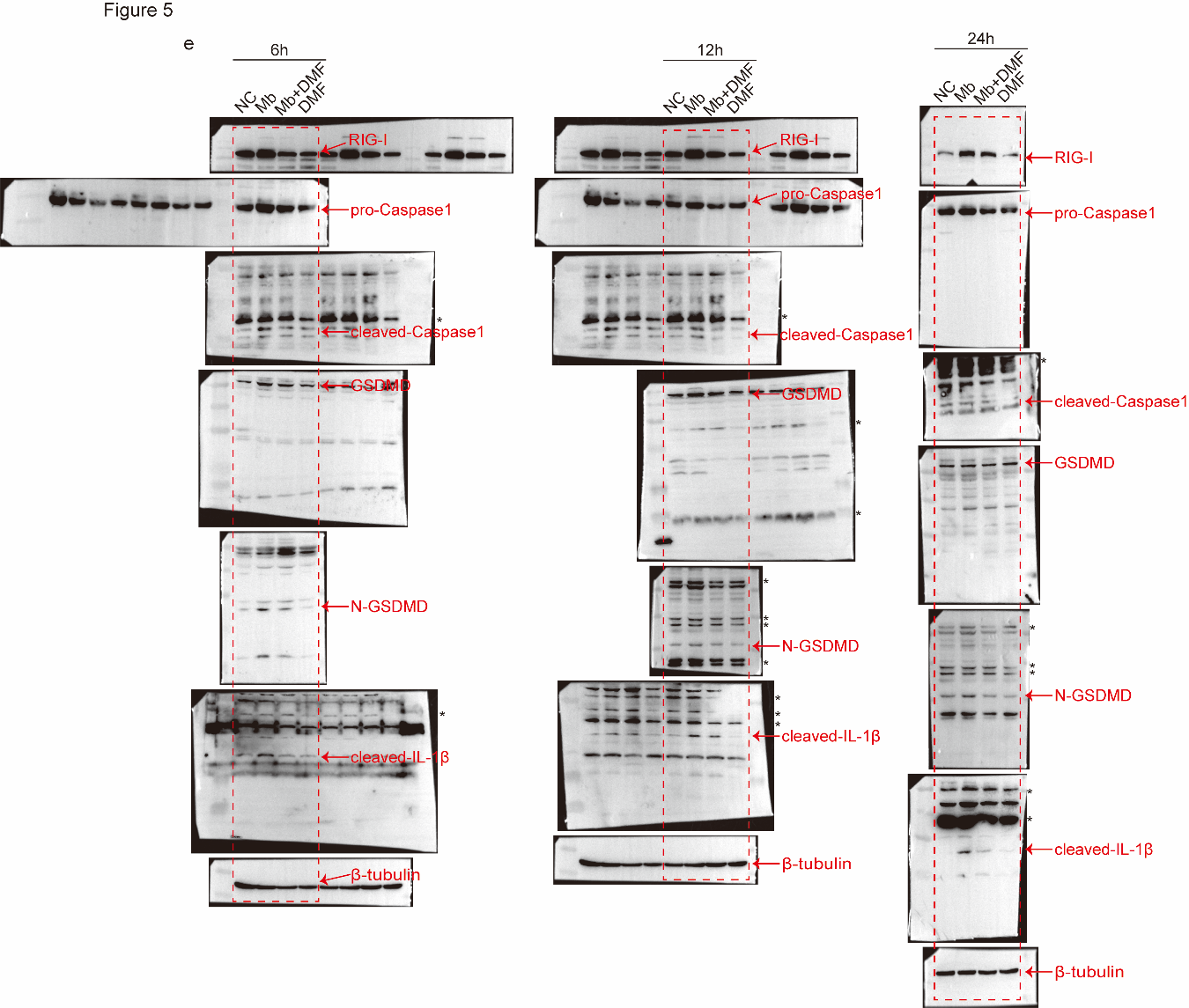


Notes：Gray asterisks denote a non-specific band.

**The original WB image of Figure 6**


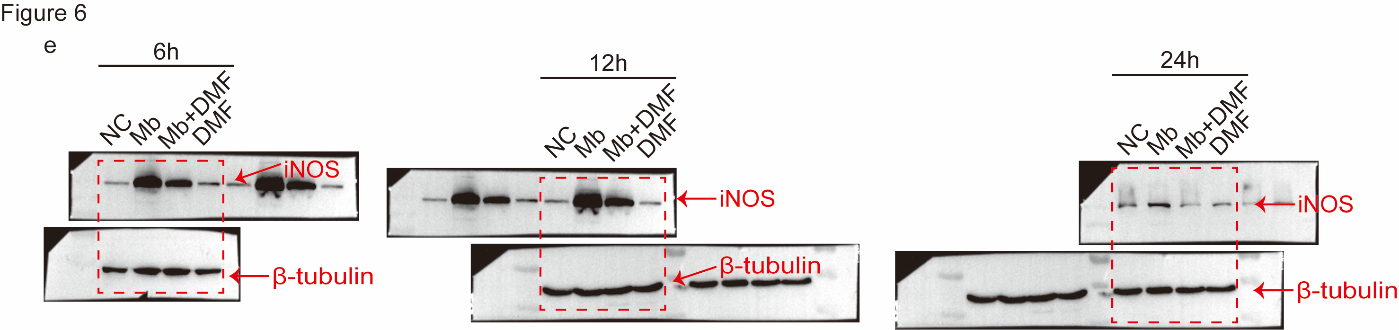

Supplement: Supplementary file 1 — Original Data File [file 41420_2022_894_MOESM1_ESM.docx]
