# Supplementary material for: Myoglobin promotes macrophage polarization to M1 type and pyroptosis via the RIG-I/Caspase1/GSDMD signaling pathway in CS-AKI
Source: Cell Death Discov. 2022 Feb 28;8:90. doi: 10.1038/s41420-022-00894-w (PMC8885737; doi:10.1038/s41420-022-00894-w)
Supplement: Supplementary file 2 — Supplementary Information [file 41420_2022_894_MOESM2_ESM.docx]

**Supplementary Information for**

Myoglobin promotes macrophage polarization to M1 type and pyroptosis via the RIG-I/Caspase1/GSDMD signaling pathway in CS-AKI

**Supplementary Table 1. The primers used for qPCR detection.**

| Gene names | Forward primer (5′-3′) | Reverse primer (5′-3′) |
| --- | --- | --- |
| NGAL | TGGCCCTGAGTGTCATGTG | CTCTTGTAGCTCATAGATGGTGC |
| KIM-1 | GGTCTGTATTGTTGCCGAGTGGAG | GCCTTGTGGTTGTGGGTCTTGTAG |
| iNOS | GTTCTCAGCCCAACAATACAAGA | GTGGACGGGTCGATGTCAC |
| CD86 | TGTTTCCGTGGAGACGCAAG | TTGAGCCTTTGTAAATGGGCA |
| IL-6 | CCAAGAGGTGAGTGCTTCCC | CTGTTGTTCAGACTCTCTCCCT |
| Arg1 | CTCCAAGCCAAAGTCCTTAGAG | AGGAGCTGTCATTAGGGACATC |
| IL-10 | GCTCTTACTGACTGGCATGAG | CGCAGCTCTAGGAGCATGTG |
| RIG-I | AAGAGCCAGAGTGTCAGAATCT | AGCTCCAGTTGGTAATTTCTTGG |
| NLRP3 | ATTACCCGCCCGAGAAAGG | TCGCAGCAAAGATCCACACAG |
| Caspase1 | ACAAGGCACGGGACCTATG | TCCCAGTCAGTCCTGGAAATG |
| GSDMD | CCATCGGCCTTTGAGAAAGTG | ACACATGAATAACGGGGTTTCC |
| IL-1β | TGGACCTTCCAGGATGAGGACA | GTTCATCTCGGAGCCTGTAGTG |
| IL-18 | GACTCTTGCGTCAACTTCAAGG | CAGGCTGTCTTTTGTCAACGA |
| GAPDH | AGGTCGGTGTGAACGGATTTG | TGTAGACCATGTAGTTGAGGTCA |

**Supplementary Figure 1-5**

**Supplementary Figure 1**


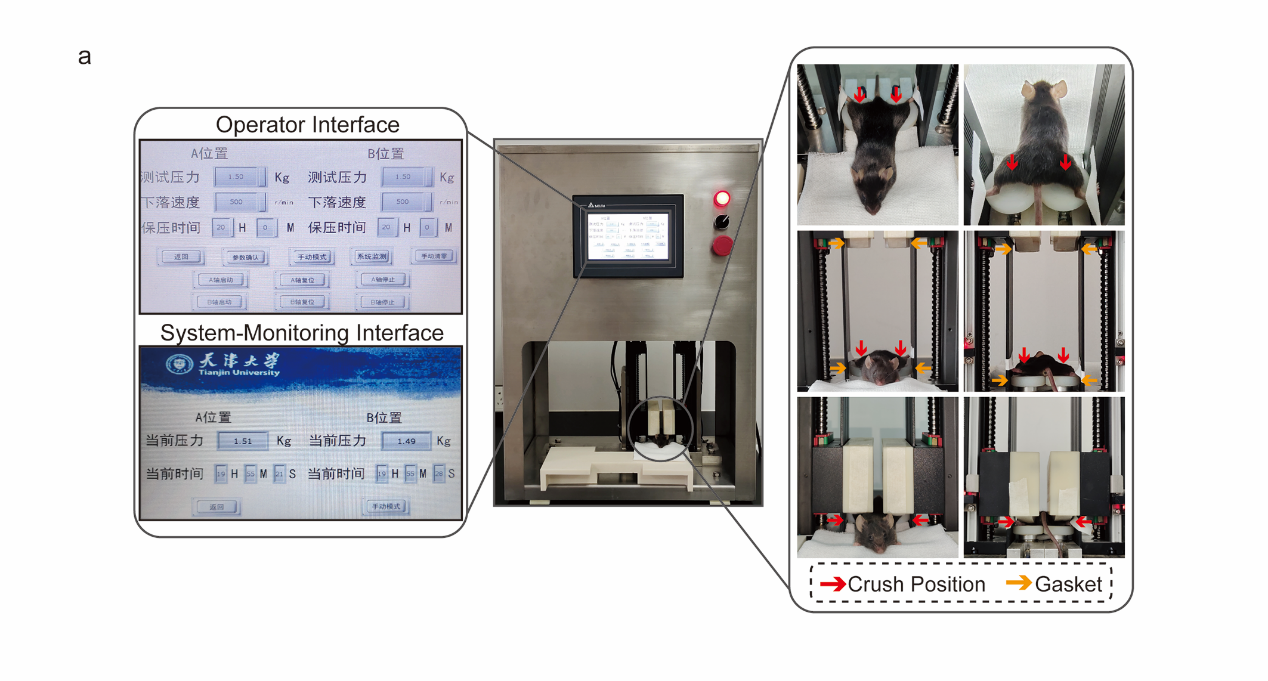


**Fig. S1. Schematic drawing of the homemade new digital crush platform.**

(a). Schematic drawing of the homemade new digital crush platform. Red arrows indicate the squeezing positions. Yellow arrows are gasket locations.

**Supplementary Figure 2**


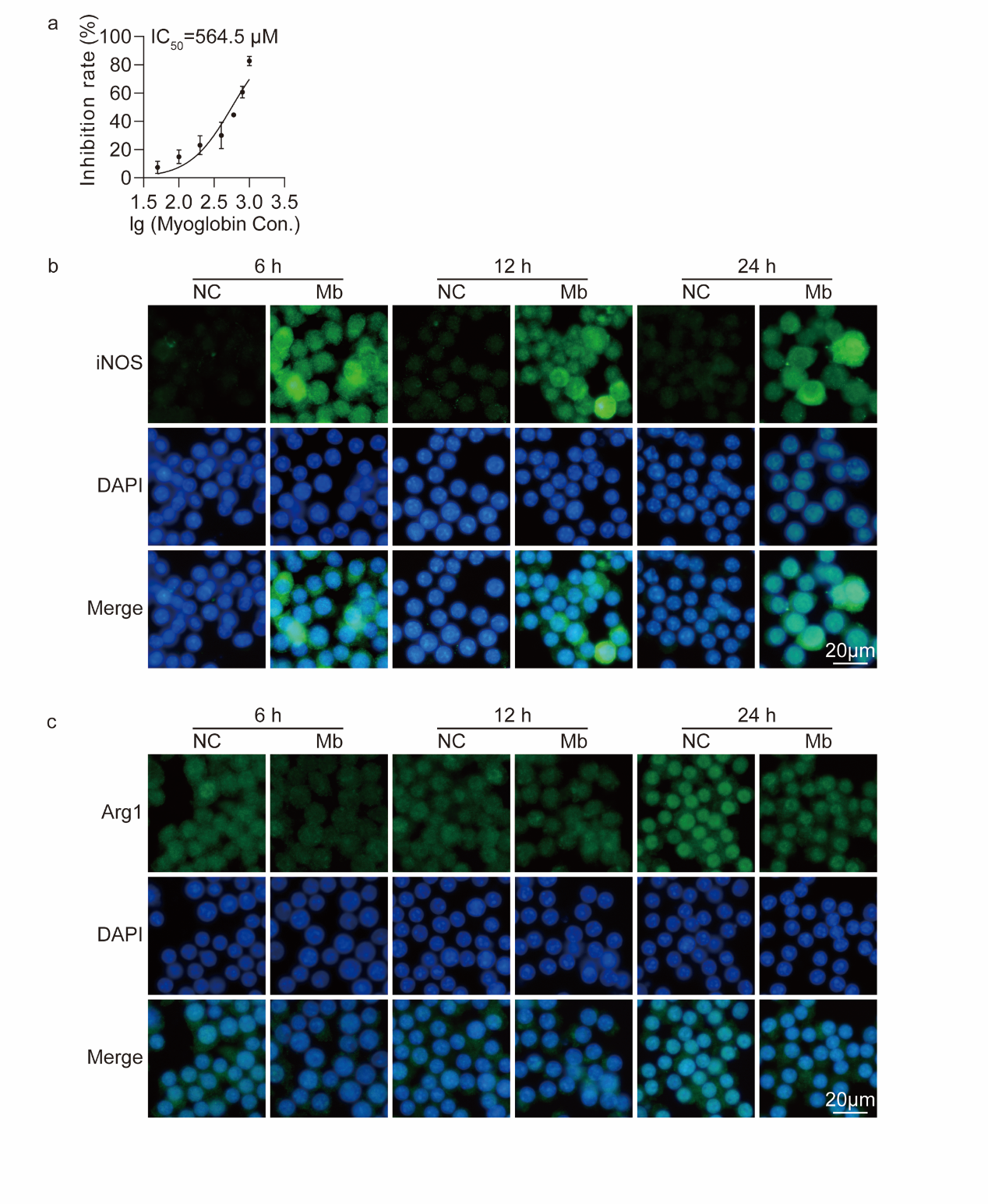


**Fig. S2. Myoglobin promotes phenotype transformation of macrophages to M1.**

(a). Inhibition rate of Raw264.7 cell growth on the treatment with 50, 100, 200, 400, 800 μM ferrous myoglobin for 6 h by CCK-8 assay. (b-c). Representative fluorescence microscope images of cells subjected to 200 μM ferrous myoglobin treatments for 6 h, 12 h and 24 h separately and stained for nuclei (DAPI, blue), anti-iNOS or anti-Arg1(green) (Scale bars: 20 μm).

**Supplementary Figure 3**


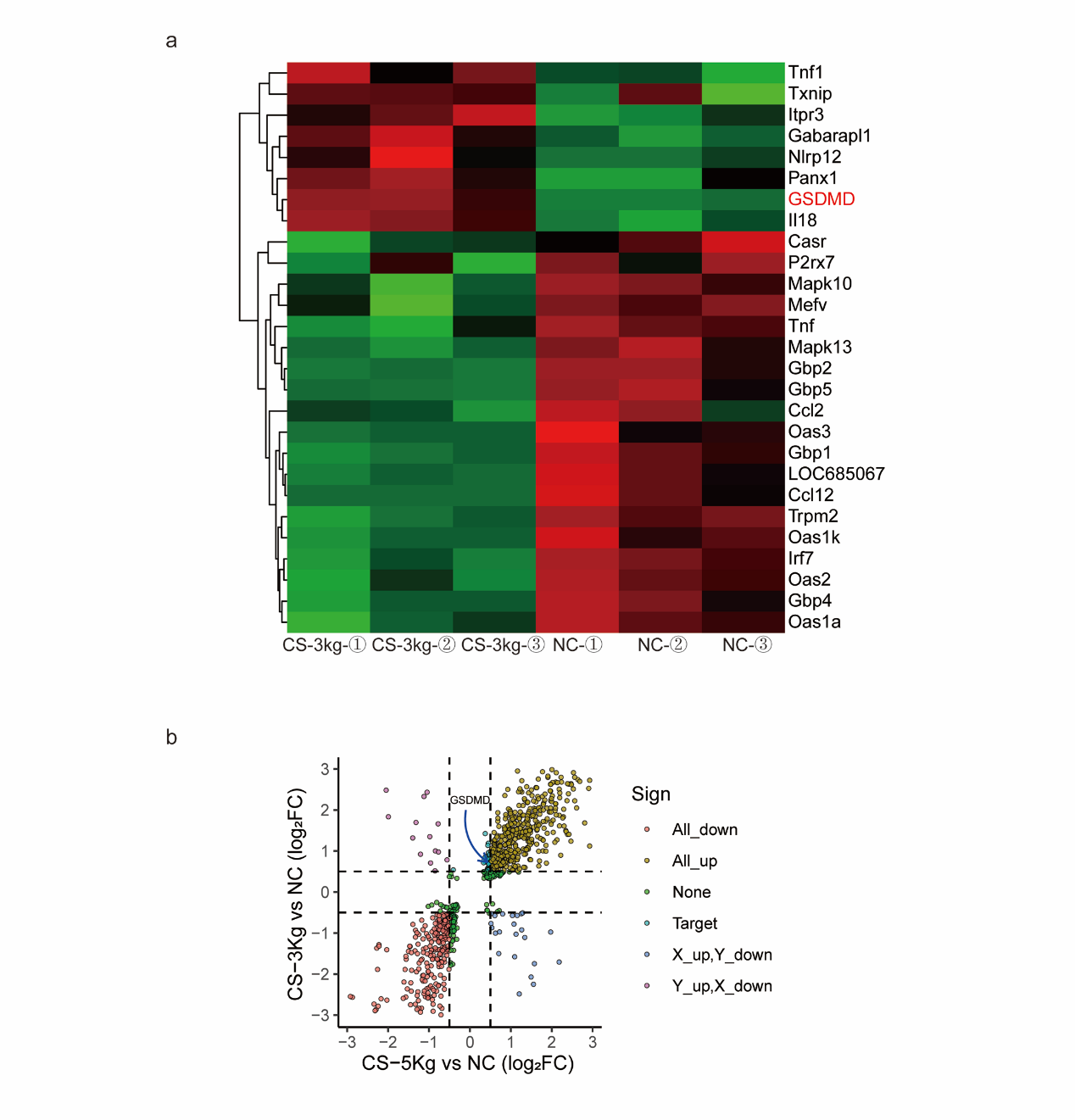


**Fig. S3. Prediction of pyroptosis pathway protein in CS-AKI by RNA Sequencing.**

(a). Heat map shows the NOD-like signaling pathway molecules in the kidney tissues of control and CS-AKI mice. (b). Dysregulated GSDMD protein in the kidney tissues of the 3 kg and 5 kg weight compression groups compared with the control group.

**Supplementary Figure 4**


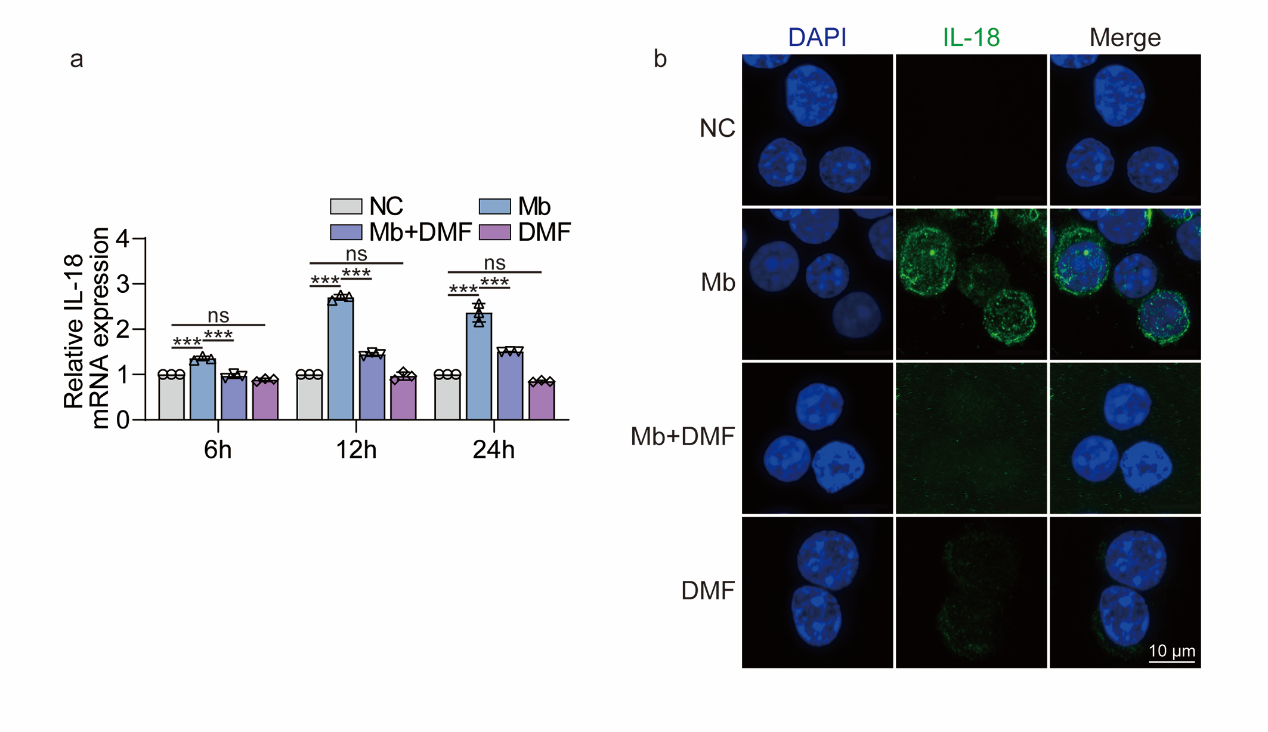


**Fig. S4. DMF decrease pyroptosis associated molecular expression.**

(a-b). qPCR and immunofluorescence analyse IL-18 expression in NC, Mb, Mb+DMF, and DMF group. For statistical analysis, two-factor ANOVA followed by Tukey's method of multiple comparisons used in (a). Data are expressed as mean ± SD, n=3 per group. *** *P* < 0.001.

**Supplementary Figure 5**


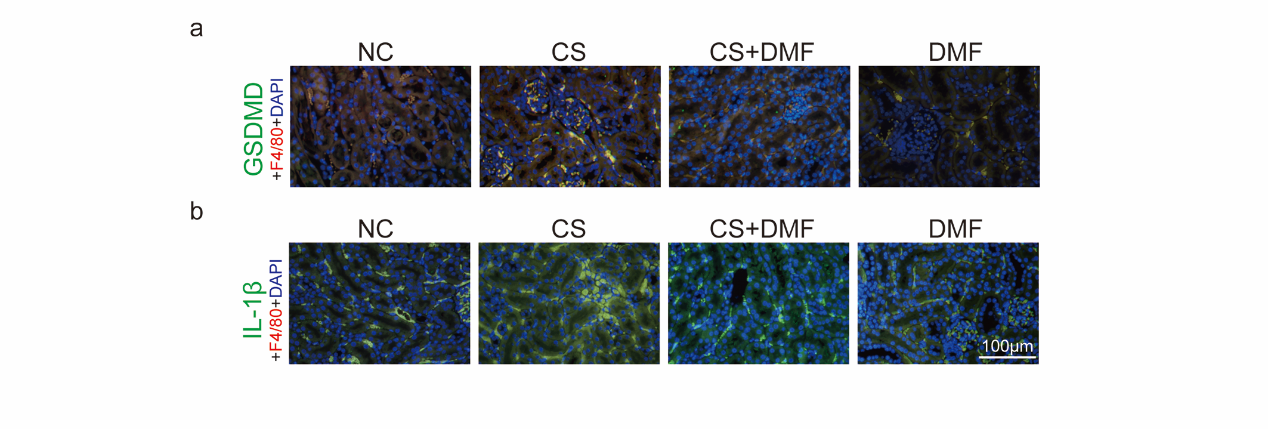


**Fig. S5 DMF reduces macrophage pyroptosis molecules expression in the kidney of CS-AKI mice.**

(a-b). Representative confocal microscopy images of sections from kidneys harvested in NC, CS, CS+DMF and DMF group mice stained for GSDMD and IL-1β (green), F4/80 (red) and DAPI (blue) (scale bar: 100 μm). n = 6.
